# Urinary pesticide biomarkers from adolescence to young adulthood in an agricultural setting in Ecuador: Study of secondary exposure to pesticides among children, adolescents, and adults (ESPINA) 2016 and 2022 examination data

**DOI:** 10.1016/j.dib.2025.111882

**Published:** 2025-07-11

**Authors:** Rajendra P. Parajuli, Briana N.C. Chronister, Dana Boyd Barr, José Ricardo Suárez-López

**Affiliations:** a Climate and Environmental Health Research Program, Herbert Wertheim School of Public Health and Human Longevity Science, University of California, San Diego. 9500 Gilman Drive #0725, La Jolla, CA 92093-0725, USA; b Gangarosa Department of Environmental Health, Rollins School of Public Health, Emory University, 1518 Clifton Rd N E, Atlanta, GA 30322, USA

**Keywords:** Pesticide metabolite, Adolescents, Agriculture, Cohort, Ecuador

## Abstract

A widespread reliance on pesticides in agriculture has raised concerns regarding the increased risks of exposure among individuals living in agricultural communities. This article presents urinary pesticide metabolite concentrations for 665 participants in the “Study of Secondary Exposure to Pesticides among Children, Adolescents, and Adults” (ESPINA), which were collected during two follow-up assessments: July to October 2016 (Follow-up Year [FUY]-8b; *n* = 529) and July to September 2022 (FUY-14a; *n* = 505) in the agricultural community of Pedro Moncayo, Ecuador. Using first morning void urine samples, concentrations of 19 pesticides or their metabolites were measured including four organophosphates, three pyrethroids, and six neonicotinoids insecticides, two herbicides, two fungicides, and two insect repellents, for the 2016 examination (FUY-8b). In the 2022 examination (FUY-14a), fourteen biomarkers were measured, including four organophosphates, three pyrethroids, three neonicotinoids, two sulfoximines, and one butanolide insecticides, and one herbicide. We present wet weight, urinary-creatinine-adjusted concentrations (2016) and specific-gravity-adjusted concentrations (2016 and 2022).

Specifications Table

**Table:** 

Subject	Health Sciences, Medical Sciences & Pharmacology
Specific subject area	Pesticide exposure assessment in agricultural communities using urinary biomarkers
Type of data	Table Raw, Analysed, Filtered, Processed
Data collection	Surveys of parents (2016) and participants (2022) were conducted, and participant examinations were conducted in schools in Pedro Moncayo County, Ecuador at both time periods. First morning urine was collected by participants. Frozen biospecimens were shipped to the University of California San Diego and subsequently to the Division of Laboratory Science of the Centers for Disease Control and Prevention and Emory University for quantitation of pesticide biomarkers in urine.
Data source location	Location of participants: Pedro Moncayo County, Pichincha, Ecuador Latitude: 0° 12′ 60.00″ N Longitude: −78° 30′ 59.99″ W Institutions: University of California San Diego, La Jolla, CA, USA; Fundación Cimas del Ecuador, Quito, Pichincha, Ecuador.
Data accessibility	Repository name: Mendeley Data identification number: 10.17632/vfvg653djb.1 Direct URL to data: https://data.mendeley.com/datasets/vfvg653djb/1 Click the above link and download data with Data Dictionary and Prefix Description
Related research article	None

## 1 Value of the Data

• This dataset provides concentrations of 19 urinary pesticide biomarkers measured in adolescents and young adults from an agricultural region in Ecuador (Pedro Moncayo) in 2016 and 2022. It offers valuable insights into exposures to organophosphates, pyrethroids, herbicides, and fungicides in an underrepresented Latin American population.
• The data are reported as both raw (µg/L) and corrected concentrations (creatinine- and specific gravity-adjusted), enabling standardized comparisons across biomonitoring studies and populations of varying age, geography, or physiological status.
• These data can be reused to evaluate temporal trends in pesticide exposure within the same population, which may reflect shifts in agricultural practices, regulatory changes, or environmental influences between 2016 and 2022.
• The dataset is suitable for pooled analyses or meta-analyses exploring pesticide exposure distributions across global populations, particularly within adolescent or agricultural community health contexts.

## 2 Background

Biomonitoring pesticide exposures in agricultural communities is crucial for understanding the real-world impacts of pesticide use on human health [1]. Unlike self-reported data or environmental sampling, biomonitoring provides objective, quantitative evidence of exposure, capturing multiple pathways such as inhalation, ingestion, and dermal absorption. This is particularly important for studying chronic, low-dose exposures, which are common in agricultural settings and may contribute to long-term health effects, including neurodevelopmental, reproductive, and endocrine disruptions [2]. Furthermore, characterizing exposures in high-risk and vulnerable groups, such as children and adolescents, can allow for targeted public health interventions. In Latin America, where agricultural work is a primary livelihood, biomonitoring is especially valuable, considering the limited resources available in these settings to conduct this work [3]. Many rural communities have limited access to healthcare, making early detection of pesticide-related health risks critical. Additionally, differences in pesticide regulations, enforcement, and safety practices highlight the need for region-specific exposure assessments. Biomonitoring can also help strengthen local research capacity, inform policymakers and support regulatory efforts [4]. In the present article, we provide pesticide biomonitoring data at two time points of adolescents and young adults in a longitudinal cohort study in the agricultural county of Pedro Moncayo, Ecuador.

## 3 Data Description

We present data on urinary pesticide metabolites from two follow-up examinations: July to October 2016 (Follow-up Year [FUY] 8b) and July to September 2022 (FUY-14a) of participants in the Secondary Exposure to Pesticides Among Children, Adolescents, and Adults (ESPINA) study. The data in the article are distributed in one excel file containing “the data” with “data dictionary” and “prefix description” and one-word file containing the metabolite concentration summary data presented in 5 tables.

Most participants in FUY-8b were adolescents (mean age [SD]: 14.5 years [1.8], 49.3 % male) and in FUY-14a most were young adults (mean age [SD]: 20.3 years [1.8], 49.5 % male).

Tables 1, 2, 3, 4, and 5 present the geometric means (95 % confidence intervals [95 % CIs]), means (95 % CIs), percentile distributions, levels of detection (LODs), interfering substance counts (substances that precluded the measurement of the targeted chemical), and detection rates for the FUY-8b wet weights (Table 1), FUY-8b creatinine adjusted (Table 2) concentrations, FUY-8b specific gravity (Table 3) adjusted concentrations, the FUY-14a wet weights (Table 4), and the FUY-14a specific gravity (Table 5) adjusted concentrations. In FUY-14a, participants had higher urinary pesticide biomarker geometric mean concentrations for 7 biomarkers [i.e., para-nitrophenol (PNP), 3,5,6-trichloro-2-pyridinol (TCPy), 3-phenoxybenzoic acid (3-PBA), trans-3-(2,2-dichlorovinyl)-2,2-dimethylcyclopropane carboxylic acid (*trans*-DCCA), 5‑hydroxy imidacloprid (OHIM), acetamiprid-N-desmethyl (AND), clothianidin (CLOT) and lower concentrations for 2,4-D, 3-(ethylcarbamoyl) benzoic acid (ECBA) and MDA compared to FUY-8b

**Table 1:** Metabolite concentrations (Wet Weights) ug/L among adolescents of the ESPINA 2016 examination (FUY-8b; 12-17-year-olds, N=529) in Pedro Moncayo, Ecuador.

Chemical class	Metabolite	Parent chemical	LOD	Interf. Subst. (n)	% Detectable	%Detectable +%Trace Conc.^a^	Range µg/L	Geometric Mean µg/L (95% CI)	Percentile cutoffs, µg/L (95% CI)					N#
									25%	50%	75%	90%	95%	
Organophosphate	PNP	Parathion, Methyl parathion	0.10	5	100.0%	100.0%	0.11 - 2.57	0.52 (0.49, 0.54)	0.36 (0.34, 0.38)	0.50 (0.47, 0.53)	0.73 (0.67, 0.78)	1.12 (0.96, 1.29)	1.45 (1.25, 1.65)	523
	TCPy	Chlorpyrifos, Chlorpyrifos-methyl	0.10	0	100.0%	100.0%	0.28 - 47.60	2.68 (2.50, 2.87)	1.55 (1.42, 1.68)	2.68 (2.49, 2.87)	4.48 (4.06, 4.90)	6.95 (6.23, 7.67)	9.50 (7.43,11.57)	528
	IMPy	Diazinon	0.10	3	23.8%	23.8%	0.07 - 6.67	*	<LOD	<LOD	<LOD	0.28 (0.21, 0.35)	0.55 (0.36, 0.74)	525
	MDA	Malathion	0.50	0	37.7%	37.7%	0.35 - 18.30	0.50 (0.48, 0.52)	<LOD	<LOD	0.77 (0.74, 0.79)	0.92 (0.88, 0.97)	1.04 (0.90, 1.18)	528
Pyrethroid	3-PBA	Cyhalothrin, Cypermethrin, Deltamethrin, Fenpropathrin, Permethrin, Tralomethrin	0.10	26	88.6%	88.7%	0.07 - 14.20	0.39 (0.35, 0.42)	0.21 (0.19, 0.24)	0.37 (0.33, 0.41)	0.68 (0.59, 0.78)	1.35 (1.07, 1.63)	2.30 (1.59, 3.01)	502
	4F-3-PBA	Cyfluthrin, Flumethrin	0.1	0	0.0%	0.0%	-	*	<LOD	<LOD	<LOD	<LOD	<LOD	528
	*trans*- DCCA	Permethrin; Cypermethrin; Cyfluthrin	0.60	12	16.1%	16.1%	0.42 - 21.30	*	<LOD	<LOD	<LOD	0.99 (0.68, 1.30)	1.96 (1.20, 2.71)	516
Neonicotinoids	OHIM	Imidacloprid	0.40	41	28.5%	28.5%	0.28 - 17.60	*	<LOD	<LOD	0.48 (0.31, 0.65)	1.13 (0.91, 1.34)	1.44 (0.92, 1.96)	487
	AND	Acetamiprid	0.20	18	37.8%	37.8%	0.14 - 22.80	0.26 (0.24, 0.29)	<LOD	<LOD	0.45 (0.36, 0.54)	0.99 (0.67, 1.31)	2.47 (1.54, 3.40)	510
	IMID	Imidacloprid	0.40	10	7.7%	7.7%	0.28 - 1.15	*	<LOD	<LOD	<LOD	<LOD	0.48 (0.42, 0.54)	518
	CLOT	Clothianidin, Thiamethoxam	0.20	0	2.8%	2.8%	0.14 - 8.87	*	<LOD	<LOD	<LOD	<LOD	<LOD	528
	ACET	Acetamiprid	0.30	1	1.1%	1.1%	0.21 - 1.20	*	<LOD	<LOD	<LOD	<LOD	<LOD	527
	THIA	Thiacloprid	0.03	0	0.0%	0.0%	-	*	<LOD	<LOD	<LOD	<LOD	<LOD	528
Herbicide	2,4-D	2,4-Dichlorophenoxy-acetic acid (and it's esters)	0.15	0	66.3%	66.3%	0.11 - 69.70	0.24 (0.22, 0.25)	<LOD	0.21 (0.19, 0.23)	0.37 (0.34, 0.40)	0.62 (0.51, 0.72)	1.00 (0.75, 1.25)	528
	GLY	Glyphosate	0.25	0	87.0%	100.0%	0.00 - 23.70	0.74 (0.64, 0.85)	0.41 (0.35, 0.46)	0.87 (0.77, 0.97)	1.79 (1.54, 2.03)	3.45 (3.02, 3.89)	4.70 (3.84, 5.57)	529
Fungicide	PTU	Ethylene bis-dithiocarbamates (EBDC)	1.63	0	10.2%	72.2%	0.16 - 36.30	0.82 (0.77, 0.87)	<LOD	1.00 (0.84, 1.15)	1.15 (1.06, 1.23)	1.67 (1.41, 1.93)	2.73 (1.88, 3.57)	529
	ETU	Ethylene bis-dithiocarbamates (EBDC)	0.63	0	81.3%	90.4%	0.28 - 27.30	1.45 (1.35, 1.56)	0.79 (0.69, 0.89)	1.45 (1.35, 1.54)	2.36 (2.14, 2.58)	4.05 (3.55, 4.56)	5.84 (3.93, 7.74)	529
DEET Insect repellent	DCBA	N,N-diethyl-meta-toluamide (DEET)	0.20	7	62.4%	62.4%	0.14 - 1840	0.55 (0.47, 0.63)	<LOD	0.31 (0.26, 0.37)	1.06 (0.68, 1.45)	6.22 (3.70, 8.74)	18.84 (9.65,28.04)	521
	ECBA	N,N-diethyl-meta-toluamide (DEET)	0.20	0	34.1%	34.1%	0.14 - 907	0.32 (0.28, 0.36)	<LOD	<LOD	0.38 (0.21, 0.54)	3.00 (1.57, 4.43)	11.08 (5.20,16.96)	528

FUY8b: Follow-Up Year 8 "b" for ESPINA Jul-Oct 2016 examination; Interf. Subst.=Interfering substances; LOD= limit of detection. Conc.= Concentration

PNP: para-Nitrophenol; TCPy: 3,5,6-Trichloro-2-pyridinol; IMPy: 2-isopropyl-4-methyl-6-hydroxypyrimidine; MDA: Malathion dicarboxylic acid; 3-PBA: 3-phenoxybenzoic acid; 4F-3-PBA: 4-fluoro-3-phenoxybenzoic acid; trans-DCCA: trans-3-(2,2-Dichlorovinyl)-2,2-dimethylcyclopropane carboxylic acid; OHIM: 5-Hydroxy imidacloprid; AND: Acetamiprid-N-desmethyl; IMID: Imidacloprid; CLOT: Clothianidin; ACET: Acetamiprid; THIA: Thiacloprid; 2,4-D: 2,4-Dichlorophenoxy-acetic acid; GLY: Glyphosate; PTU: Propylene thiourea; ETU: Ethylene thiourea; DCBA: 3-(diethylcarbamoyl)benzoic acid; ECBA: 3-(ethylcarbamoyl)benzoic acid.

Values below LOD were assigned LOD/sqrt (2).

# Number of samples calculated by subtracting observations with interfering substances from measured samples.

⁎ If the proportion of results below the LOD was greater than 70%, geometric means were not calculated.

a Glyphosate, ETU, and PTU had machine-derived trace concentrations quantified, even though they were below the LOD.

**Table 2:** Metabolite concentrations (ug/g of creatinine) among adolescents of the ESPINA 2016 examination (FUY-8b; 12-17-year-olds, N=529) in Pedro Moncayo, Ecuador.

Chemical class	Metabolite	Parent chemical	LOD	Interf. Subst. (n)	% Detectable	%Detectable +%Trace Conc.^a^	Range µg/g	Geometric Mean µg/g (95% CI)	Percentile cutoffs, µg/g (95% CI)					N#
									25%	50%	75%	90%	95%	
Organophosphate	PNP	Parathion, Methyl parathion	0.10	5	100.0%	100.0%	0.14 - 2.63	0.59 (0.57, 0.62)	0.45 (0.42, 0.47)	0.58 (0.56, 0.60)	0.80 (0.76, 0.83)	1.07 (1.00, 1.15)	1.35 (1.12, 1.58)	523
	TCPy	Chlorpyrifos, Chlorpyrifos-methyl	0.10	0	100.0%	100.0%	0.42 - 29.57	3.07 (2.89, 3.26)	1.81 (1.69, 1.93)	2.82 (2.63, 3.01)	4.66 (4.19, 5.12)	8.02 (6.76, 9.28)	11.00 (8.79,13.20)	528
	IMPy	Diazinon	0.10	3	23.8%	23.8%	0.02 - 3.72	*	<LOD	<LOD	<LOD	0.33 (0.28, 0.38)	0.52 (0.21, 0.84)	525
	MDA	Malathion	0.50	0	37.7%	37.7%	0.09 - 14.08	*	<LOD	<LOD	0.92 (0.84, 0.99)	1.40 (1.25, 1.55)	1.79 (1.58, 2.00)	528
Pyrethroid	3-PBA	Cyhalothrin, Cypermethrin, Deltamethrin, Fenpropathrin, Permethrin, Tralomethrin	0.10	26	88.6%	88.6%	0.04 - 13.3	0.45 (0.42, 0.49)	0.26 (0.23, 0.29)	0.44 (0.41, 0.47)	0.74 (0.67, 0.82)	1.42 (1.13, 1.71)	2.22 (1.74, 2.70)	502
	4F-3-PBA	Cyfluthrin, Flumethrin	0.10	0	0.0%	0.0%	-	*	<LOD	<LOD	<LOD	<LOD	<LOD	528
	*trans-*DCCA	Permethrin; Cypermethrin; Cyfluthrin	0.60	12	16.1%	16.1%	0.11 - 9.34	*	<LOD	<LOD	<LOD	1.58 (1.38, 1.79)	2.34 (1.76, 2.92)	516
Neonicotinoids	OHIM	Imidacloprid	0.40	41	28.5%	28.5%	0.07 - 28.09	*	<LOD	<LOD	0.77 (0.69, 0.85)	1.29 (1.12, 1.47)	1.88 (1.21, 2.54)	487
	AND	Acetamiprid	0.20	18	37.8%	37.8%	0.04 - 44.79	0.31 (0.28, 0.34)	<LOD	<LOD	0.54 (0.46, 0.62)	1.23 (0.75, 1.71)	2.85 (1.88, 3.82)	510
	IMID	Imidacloprid	0.40	10	7.7%	7.7%	0.07 - 2.68	*	<LOD	<LOD	<LOD	<LOD	1.02 (0.87, 1.16)	518
	CLOT	Clothianidin, Thiamethoxam	0.20	0	2.8%	2.8%	0.04 - 4.47	*	<LOD	<LOD	<LOD	<LOD	<LOD	528
	ACET	Acetamiprid	0.30	1	1.1%	1.1%	0.05 - 1.67	*	<LOD	<LOD	<LOD	<LOD	<LOD	527
	THIA	Thiacloprid	0.03	0	0.0%	0.0%	-	*	<LOD	<LOD	<LOD	<LOD	<LOD	528
Herbicide	2,4-D	2,4-Dichlorophenoxy-acetic acid (and it's esters)	0.15	0	66.3%	66.3%	0.04 - 57.6	0.27 (0.25, 0.29)	<LOD	0.25 (0.23, 0.27)	0.41 (0.37, 0.45)	0.70 (0.59, 0.81)	0.98 (0.76, 1.20)	528
	GLY	Glyphosate	0.25	0	87.0%	100.0%	0 - 21.94	0.84 (0.74, 0.96)	0.53 (0.47, 0.60)	1.00 (0.90, 1.11)	1.93 (1.75, 2.11)	3.40 (3.01, 3.79)	4.83 (4.10, 5.56)	529
Fungicide	PTU	Ethylene bis-dithiocarbamates (EBDC)	1.63	0	10.2%	72.2%	0.07 - 104.01	0.94 (0.87, 1.01)	<LOD	0.94 (0.86, 1.02)	1.61 (1.43, 1.79)	2.74 (2.37, 3.12)	4.02 (3.33, 4.71)	529
	ETU	Ethylene bis-dithiocarbamates (EBDC)	0.63	0	81.3%	90.4%	0.17 - 37.06	1.66 (1.54, 1.79)	0.89 (0.79, 0.98)	1.70 (1.55, 1.84)	2.91 (2.60, 3.21)	4.57 (3.93, 5.21)	6.64 (4.94, 8.33)	529
DEET Insect repellent	DCBA	N,N-diethyl-meta-toluamide (DEET)	0.20	7	62.4%	62.4%	0.04 - 1612	0.63 (0.55, 0.73)	<LOD	0.39 (0.34, 0.44)	1.13 (0.86, 1.41)	5.78 (3.24, 8.33)	16.73 (5.97,27.48)	521
	ECBA	N,N-diethyl-meta-toluamide (DEET)	0.20	0	34.1%	34.1%	0.04 - 750	0.36 (0.32, 0.41)	<LOD	<LOD	0.55 (0.45, 0.64)	2.98 (1.41, 4.55)	9.36 (3.49,15.23)	528

FUY8b: Follow-Up Year 8 "b" for ESPINA Jul-Oct 2016 examination; Interf. Subst.=Interfering substances; LOD= limit of detection. Conc.= Concentration

PNP: para-Nitrophenol; TCPy: 3,5,6-Trichloro-2-pyridinol; IMPy: 2-isopropyl-4-methyl-6-hydroxypyrimidine; MDA: Malathion dicarboxylic acid; 3-PBA: 3-phenoxybenzoic acid; 4F-3-PBA: 4-fluoro-3-phenoxybenzoic acid; trans-DCCA: trans-3-(2,2-Dichlorovinyl)-2,2-dimethylcyclopropane carboxylic acid; OHIM: 5-Hydroxy imidacloprid; AND: Acetamiprid-N-desmethyl; IMID: Imidacloprid; CLOT: Clothianidin; ACET: Acetamiprid; THIA: Thiacloprid; 2,4-D: 2,4-Dichlorophenoxy-acetic acid; GLY: Glyphosate; PTU: Propylenethiourea; ETU: Ethylenethiourea; DCBA: 3-(diethylcarbamoyl)benzoic acid; ECBA: 3-(ethylcarbamoyl)benzoic acid.

Values below LOD were assigned LOD/sqrt (2).

# Number of samples calculated by subtracting observations with interfering substances from measured samples.

⁎ If the proportion of results below the LOD was greater than 70%, geometric means were not calculated.

a Glyphosate, ETU, and PTU had machine-derived trace concentrations quantified, even though they were below the LOD.

**Table 3:** Metabolite concentrations ug/L (adjusted for specific gravity±) among adolescents of the ESPINA 2016 examination (FUY-8b; 12-17-year-olds, N=529) in Pedro Moncayo, Ecuador.

Chemical class	Metabolite	Parent chemical	LOD	Interf. Subst. (n)	% Detectable	%Detectable +%Trace Conc.^a^	Range µg/L	Geometric Mean µg/L (95% CI)	Percentile cutoffs, µg/L (95% CI)					N#
									25%	50%	75%	90%	95%	
Organophosphate	PNP	Parathion, Methyl parathion	0.10	5	100.0%	100.0%	0.19 - 2.04	0.54 (0.52, 0.56)	0.40 (0.38, 0.42)	0.52 (0.49, 0.54)	0.69 (0.66, 0.73)	0.96 (0.87, 1.06)	1.27 (1.10, 1.43)	523
	TCPy	Chlorpyrifos, Chlorpyrifos-methyl	0.10	0	100.0%	100.0%	0.49 - 36.87	2.79 (2.63, 2.96)	1.72 (1.61, 1.82)	2.70 (2.48, 2.92)	4.34 (3.93, 4.76)	6.85 (6.09, 7.62)	9.69 (7.85,11.52)	528
	IMPy	Diazinon	0.10	3	23.8%	23.8%	0.05 - 5.05	*	<LOD	<LOD	<LOD	0.27 (0.21, 0.32)	0.50 (0.26, 0.74)	525
	MDA	Malathion	0.50	0	37.7%	37.7%	0.26 - 14.33	0.52 (0.50, 0.55)	<LOD	<LOD	0.76 (0.72, 0.79)	1.01 (0.93, 1.09)	1.29 (1.07, 1.51)	528
Pyrethroid	3-PBA	Cyhalothrin, Cypermethrin, Deltamethrin, Fenpropathrin, Permethrin, Tralomethrin	0.10	26	88.6%	88.6%	0.06 - 11.22	0.40 (0.37, 0.44)	0.23 (0.21, 0.25)	0.38 (0.35, 0.41)	0.69 (0.62, 0.75)	1.29 (1.04, 1.54)	2.09 (1.63, 2.55)	502
	4F-3-PBA	Cyfluthrin, Flumethrin	0.10	0	0.0%	0.0%	-	*	<LOD	<LOD	<LOD	<LOD	<LOD	528
	*trans-*DCCA	Permethrin; Cypermethrin; Cyfluthrin	0.60	12	16.1%	16.1%	0.31 - 15.89	*	<LOD	<LOD	<LOD	1.08 (0.87, 1.29)	1.72 (1.11, 2.32)	516
Neonicotinoids	OHIM	Imidacloprid	0.40	41	28.5%	28.5%	0.21 - 21.42	*	<LOD	<LOD	0.60 (0.54, 0.65)	1.03 (0.89, 1.16)	1.69 (1.18, 2.20)	487
	AND	Acetamiprid	0.20	18	37.8%	37.8%	0.10 - 34.15	0.28 (0.25, 0.30)	<LOD	<LOD	0.42 (0.33, 0.50)	1.13 (0.75, 1.51)	2.34 (1.20, 3.47)	510
	IMID	Imidacloprid	0.40	10	7.7%	7.7%	0.21 - 1.72	*	<LOD	<LOD	<LOD	<LOD	0.60 (0.55, 0.64)	518
	CLOT	Clothianidin, Thiamethoxam	0.20	0	2.8%	2.8%	0.10 - 6.59	*	<LOD	<LOD	<LOD	<LOD	<LOD	528
	ACET	Acetamiprid	0.30	1	1.1%	1.1%	0.15 - 1.25	*	<LOD	<LOD	<LOD	<LOD	<LOD	527
	THIA	Thiacloprid	0.03	0	0.0%	0.0%	-	*	<LOD	<LOD	<LOD	<LOD	<LOD	528
Herbicide	2,4-D	2,4-Dichlorophenoxy-acetic acid (and it's esters)	0.15	0	66.3%	66.3%	0.08 - 60.85	0.24 (0.23, 0.26)	<LOD	0.23 (0.21, 0.25)	0.35 (0.32, 0.39)	0.59 (0.46, 0.72)	0.95 (0.72, 1.18)	528
	GLY	Glyphosate	0.25	0	87.0%	100.0%	0.00 - 18.36	0.77 (0.67, 0.88)	0.46 (0.40, 0.51)	0.91 (0.81, 1.01)	1.78 (1.54, 2.03)	3.17 (2.67, 3.67)	4.20 (3.77, 4.64)	529
Fungicide	PTU	Ethylene bis-dithiocarbamates (EBDC)	1.63	0	10.2%	72.2%	0.15 - 61.56	0.85 (0.80, 0.91)	<LOD	0.89 (0.84, 0.93)	1.32 (1.20, 1.43)	1.95 (1.68, 2.22)	2.82 (2.29, 3.34)	529
	ETU	Ethylene bis-dithiocarbamates (EBDC)	0.63	0	81.3%	90.4%	0.26 - 30.06	1.51 (1.41, 1.62)	0.84 (0.75, 0.93)	1.49 (1.38, 1.59)	2.45 (2.30, 2.60)	3.95 (3.46, 4.45)	5.56 (4.33, 6.79)	529
DEET Insect repellent	DCBA	N,N-diethyl-meta-toluamide (DEET)	0.20	7	62.4%	62.4%	0.10 - 1551	0.57 (0.50, 0.66)	<LOD	0.32 (0.29, 0.35)	1.16 (0.84, 1.48)	5.90 (3.59, 8.21)	19.82 (10.12,29.52)	521
	ECBA	N,N-diethyl-meta-toluamide (DEET)	0.20	0	34.1%	34.1%	0.10 - 765	0.33 (0.29, 0.37)	<LOD	<LOD	0.42 (0.27, 0.56)	2.83 (1.44, 4.22)	10.50 (4.86,16.14)	528

FUY8b: Follow-Up Year 8 "b" for ESPINA Jul-Oct 2016 examination; Interf. Subst.=Interfering substances; LOD= limit of detection. Conc.= Concentration

PNP: para-Nitrophenol; TCPy: 3,5,6-Trichloro-2-pyridinol; IMPy: 2-isopropyl-4-methyl-6-hydroxypyrimidine; MDA: Malathion dicarboxylic acid; 3-PBA: 3-phenoxybenzoic acid; 4F-3-PBA: 4-fluoro-3-phenoxybenzoic acid; trans-DCCA: trans-3-(2,2-Dichlorovinyl)-2,2-dimethylcyclopropane carboxylic acid; OHIM: 5-Hydroxy imidacloprid; AND: Acetamiprid-N-desmethyl; IMID: Imidacloprid; CLOT: Clothianidin; ACET: Acetamiprid; THIA: Thiacloprid; 2,4-D: 2,4-Dichlorophenoxy-acetic acid; GLY: Glyphosate; PTU: Propylenethiourea; ETU: Ethylenethiourea; DCBA: 3-(diethylcarbamoyl)benzoic acid; ECBA: 3-(ethylcarbamoyl)benzoic acid.

Values below LOD were assigned LOD/sqrt (2). ^±^Specific gravity was directly measured in 2022 (FUY-14) but imputed from creatinine concentrations in 2016 (FUY-8) to enable comparison across time points.

# Number of samples calculated by subtracting observations with interfering substances from measured samples.

⁎ If the proportion of results below the LOD was greater than 70%, geometric means were not calculated.

a Glyphosate, ETU, and PTU had machine-derived trace concentrations quantified, even though they were below the LOD.

**Table 4:** Metabolite concentrations (Wet Weights) ug/L among adolescents and adults of the ESPINA 2022 examination (FUY-14a; 17-23-year-olds, N=505) in Pedro Moncayo, Ecuador.

Chemical class	Metabolite	Parent chemical	LOD	Interf. Subst. (n)	% Detectable	Range µg/L	Geometric Mean µg/L (95% CI)	Percentile cutoffs, µg/L (95% CI)					N#
								25%	50%	75%	90%	95%	
Organophosphate	PNP	Parathion, Methyl parathion	0.10	0	100.0%	0.10 - 23.00	0.63 (0.60, 0.67)	0.41 (0.38, 0.43)	0.63 (0.59, 0.67)	0.97 (0.92, 1.02)	1.34 (1.23, 1.46)	1.60 (1.48, 1.71)	505
	TCPy	Chlorpyrifos, Chlorpyrifos-methyl	0.10	0	100.0%	0.20 - 35.00	3.39 (3.16, 3.63)	2.04 (1.81, 2.27)	3.27 (2.97, 3.57)	5.57 (5.08, 6.06)	8.85 (7.95, 9.75)	12.69 (9.60,15.77)	505
	IMPy	Diazinon	0.10	0	28.9%	0.07 - 13.00	*	<LOD	<LOD	0.12 (0.08, 0.15)	0.44 (0.30, 0.57)	0.86 (0.67, 1.05)	505
	MDA	Malathion	0.50	0	8.9%	0.35 - 12.00	*	<LOD	<LOD	<LOD	<LOD	0.66 (0.53, 0.79)	505
Pyrethroid	3-PBA	Cyhalothrin, Cypermethrin, Deltamethrin, Fenpropathrin, Permethrin, Tralomethrin	0.10	1	98.6%	0.07 - 14.00	0.51 (0.48, 0.55)	0.30 (0.28, 0.33)	0.49 (0.45, 0.53)	0.84 (0.75, 0.94)	1.37 (1.14, 1.59)	1.90 (1.56, 2.23)	504
	4F-3-PBA	Cyfluthrin, Flumethrin	0.50	0	0.6%	0.07 - 0.80	*	<LOD	<LOD	<LOD	<LOD	<LOD	505
	*trans-* DCCA	Permethrin; Cypermethrin; Cyfluthrin	0.60	0	42.2%	0.42 - 7.80	0.66 (0.62, 0.69)	<LOD	<LOD	0.92 (0.83, 1.01)	1.58 (1.36, 1.79)	2.44 (1.82, 3.05)	505
Neonicotinoid	OHIM	Imidacloprid	0.10	28	92.9%	0.07 - 28.00	0.56 (0.50, 0.62)	0.24 (0.21, 0.27)	0.50 (0.42, 0.58)	1.22 (1.07, 1.36)	2.54 (2.00, 3.07)	4.17 (2.95, 5.40)	477
	AND	Acetamiprid	0.15	10	95.2%	0.04 - 13.10	0.35 (0.32, 0.39)	0.15 (0.13, 0.17)	0.32 (0.29, 0.36)	0.66 (0.54, 0.78)	1.79 (1.42, 2.15)	2.95 (2.24, 3.65)	495
	CLOT	Clothianidin, Thiamethoxam	0.10	154	74.6%	0.07 - 5.89	0.34 (0.30, 0.39)	<LOD	0.39 (0.32, 0.45)	0.83 (0.73, 0.92)	1.35 (1.17, 1.53)	1.71 (1.46, 1.97)	351
Herbicide	2,4-D	2,4-Dichlorophenoxy-acetic acid (and it's esters)	0.15	0	80.6%	0.11 - 9.70	0.28 (0.26, 0.30)	0.16 (0.13, 0.19)	0.26 (0.24, 0.28)	0.41 (0.38, 0.44)	0.74 (0.55, 0.92)	1.34 (1.03, 1.66)	505
DEET Insect Repellent	DCBA	N,N-diethyl-meta-toluamide (DEET)	0.10	0	86.5%	0.07 - 13,800	0.59 (0.51, 0.68)	0.19 (0.15, 0.22)	0.48 (0.39, 0.57)	1.45 (1.23, 1.67)	4.72 (3.23, 6.21)	9.13 (4.07,14.19)	505
	ECBA	N,N-diethyl-meta-toluamide (DEET)	0.10	3	60.0%	0.07 - 6,330	0.26 (0.23, 0.30)	<LOD	0.18 (0.13, 0.22)	0.58 (0.47, 0.69)	2.19 (1.41, 2.96)	3.85 (1.79, 5.91)	502
Sulfoximine	SLF1	Sulfoxaflor	0.05	12	37.7%	0.04 - 8.99	0.07 (0.06, 0.08)	<LOD	<LOD	0.11 (0.08, 0.13)	0.31 (0.15, 0.48)	0.88 (0.62, 1.14)	493
	SLF2	Sulfoxaflor	0.05	1	37.3%	0.04 - 7.42	0.07 (0.06, 0.07)	<LOD	<LOD	0.10 (0.07, 0.13)	0.33 (0.18, 0.48)	0.77 (0.59, 0.95)	504
Butenolide	FLUP	Flupyradifurone	0.05	3	4.2%	0.04 - 1.25	*	<LOD	<LOD	<LOD	<LOD	<LOD	502

FUY14a: Follow-Up Year 14 "a" for ESPINA Jul-Sep 2022 examination; Yrs=Years; Interf. Subst.=Interfering substances; LOD= limit of detection.

PNP: para-Nitrophenol; TCPy: 3,5,6-Trichloro-2-pyridinol; IMPy: 2-isopropyl-4-methyl-6-hydroxypyrimidine; MDA: Malathion dicarboxylic acid; 3-PBA: 3-phenoxybenzoic acid; 4F-3-PBA: 4-fluoro-3-phenoxybenzoic acid; trans-DCCA: trans-3-(2,2-Dichlorovinyl)-2,2-dimethylcyclopropane carboxylic acid; OHIM: Imidacloprid-5-hydroxy; AND: Acetamiprid-N-desmethyl; CLOT: Clothianidin; 2,4-D: 2,4-Dichlorophenoxy-acetic acid; DCBA: 3-(diethylcarbamoyl)benzoic acid; ECBA: 3-(ethylcarbamoyl)benzoic acid; SLF1: Sulfoxaflor Isomer; SLF2: Sulfoxaflor Isomer 2; FLUP: Flupyradifurone.

Values below LOD were assigned LOD/sqrt (2).

# Number of samples calculated by subtracting observations with interfering substances from measured samples.

⁎ If the proportion of results below the LOD was greater than 70%, geometric means were not calculated.

**Table 5:** Metabolite concentrations (ug/L, adjusted for specific gravity) among adolescents and adults of the ESPINA 2022 examination (FUY-14a; 17-23-year-olds, N=505) in Pedro Moncayo, Ecuador.

Chemical class	Metabolite	Parent chemical	LOD	Interf. Subst. (n)	% Detectable	Range µg/L	Geometric Mean µg/L (95% CI)	Percentile cutoffs, µg/L (95% CI)					N#
								25%	50%	75%	90%	95%	
Organophosphate	PNP	Parathion, Methyl parathion	0.10	0	100.0%	0.08 - 24.37	0.66 (0.63, 0.69)	0.47 (0.43, 0.50)	0.66 (0.62, 0.70)	0.91 (0.86, 0.96)	1.18 (1.11, 1.26)	1.49 (1.31, 1.68)	505
	TCPy	Chlorpyrifos, Chlorpyrifos-methyl	0.10	0	100.0%	0.16 - 30.29	3.57 (3.36, 3.81)	2.22 (2.04, 2.40)	3.37 (3.19, 3.55)	5.47 (5.03, 5.91)	8.88 (7.58,10.19)	13.60 (11.01,16.20)	505
	IMPy	Diazinon	0.10	0	28.9%	0.04 - 9.54	*	<LOD	<LOD	0.15 (0.14, 0.16)	0.40 (0.26, 0.54)	0.86 (0.66, 1.07)	505
	MDA	Malathion	0.50	0	8.9%	0.19 - 15.26	*	<LOD	<LOD	<LOD	<LOD	0.84 (0.78, 0.91)	505
Pyrethroid	3-PBA	Cyhalothrin, Cypermethrin, Deltamethrin, Fenpropathrin, Permethrin, Tralomethrin	0.10	1	98.6%	0.08 - 10.4	0.54 (0.50, 0.57)	0.32 (0.30, 0.35)	0.51 (0.48, 0.55)	0.78 (0.70, 0.86)	1.30 (1.08, 1.52)	2.26 (1.68, 2.84)	504
	4F-3-PBA	Cyfluthrin, Flumethrin	0.50	0	0.6%	0.04 - 0.67	*	<LOD	<LOD	<LOD	<LOD	<LOD	505
	*trans-*DCCA	Permethrin; Cypermethrin; Cyfluthrin	0.60	0	42.2%	0.22 - 11.63	0.69 (0.65, 0.73)	<LOD	<LOD	1.00 (0.92, 1.08)	1.54 (1.34, 1.74)	2.10 (1.64, 2.56)	505
Neonicotinoid	OHIM	Imidacloprid	0.10	28	92.9%	0.04 - 33.37	0.59 (0.53, 0.65)	0.27 (0.24, 0.30)	0.55 (0.47, 0.63)	1.21 (1.04, 1.37)	2.56 (2.01, 3.11)	3.79 (3.08, 4.50)	477
	AND	Acetamiprid	0.15	10	95.2%	0.02 - 11.90	0.37 (0.33, 0.41)	0.17 (0.15, 0.19)	0.34 (0.30, 0.37)	0.68 (0.56, 0.80)	1.87 (1.47, 2.28)	3.08 (2.25, 3.91)	495
	CLOT	Clothianidin, Thiamethoxam	0.10	154	74.6%	0.04 - 11.23	0.37 (0.33, 0.41)	<LOD	0.43 (0.37, 0.50)	0.84 (0.75, 0.93)	1.23 (1.08, 1.38)	1.68 (1.16, 2.20)	351
Herbicide	2,4-D	2,4-Dichlorophenoxy-acetic acid (and it's esters)	0.15	0	80.6%	0.06 - 9.54	0.30 (0.28, 0.32)	0.18 (0.17, 0.19)	0.25 (0.24, 0.27)	0.42 (0.39, 0.46)	0.82 (0.62, 1.01)	1.36 (1.09, 1.62)	505
DEET Insect Repellent	DCBA	N,N-diethyl-meta-toluamide (DEET)	0.10	0	86.5%	0.05 – 16,449	0.62 (0.54, 0.71)	0.20 (0.17, 0.23)	0.53 (0.43, 0.62)	1.48 (1.18, 1.77)	4.22 (2.65, 5.78)	9.39 (5.13,13.66)	505
	ECBA	N,N-diethyl-meta-toluamide (DEET)	0.10	3	60.0%	0.04 – 7,545	0.27 (0.24, 0.31)	<LOD	0.19 (0.15, 0.23)	0.56 (0.44, 0.67)	2.02 (1.14, 2.89)	4.57 (2.55, 6.58)	502
Sulfoximine	SLF1	Sulfoxaflor	0.05	12	37.7%	0.02 - 6.59	0.07 (0.07, 0.08)	<LOD	<LOD	0.11 (0.08, 0.14)	0.38 (0.21, 0.56)	0.89 (0.62, 1.16)	493
	SLF2	Sulfoxaflor	0.05	1	37.3%	0.02 - 5.44	0.07 (0.06, 0.08)	<LOD	<LOD	0.11 (0.08, 0.14)	0.39 (0.27, 0.51)	0.83 (0.55, 1.11)	504
Butenolide	FLUP	Flupyradifurone	0.05	3	4.2%	0.02 - 1.31	*	<LOD	<LOD	<LOD	<LOD	<LOD	502

FUY14a: Follow-Up Year 14 "a" for ESPINA Jul-Sep 2022 examination; Interf. Subst.=Interfering substances; LOD= limit of detection.

PNP: para-Nitrophenol; TCPy: 3,5,6-Trichloro-2-pyridinol; IMPy: 2-isopropyl-4-methyl-6-hydroxypyrimidine; MDA: Malathion dicarboxylic acid; 3-PBA: 3-phenoxybenzoic acid; 4F-3-PBA: 4-fluoro-3-phenoxybenzoic acid; trans-DCCA: trans-3-(2,2-Dichlorovinyl)-2,2-dimethylcyclopropane carboxylic acid; OHIM: Imidacloprid-5-hydroxy; AND: Acetamiprid-N-desmethyl; CLOT: Clothianidin; 2,4-D: 2,4-Dichlorophenoxy-acetic acid; DCBA: 3-(diethylcarbamoyl)benzoic acid; ECBA: 3-(ethylcarbamoyl)benzoic acid; SLF1: Sulfoxaflor Isomer; SLF2: Sulfoxaflor Isomer 2; FLUP: Flupyradifurone.

Values below LOD were assigned LOD/sqrt (2).

# Number of samples calculated by subtracting observations with interfering substances from measured samples.

⁎ If the proportion of results below the LOD was greater than 70%, geometric means were not calculated.

## 4 Experimental Design, Materials and Methods

ESPINA is a prospective cohort established in 2008 that aims to investigate the impacts of pesticide exposure on development from childhood to adulthood in individuals living within the agricultural community of Pedro Moncayo, Pichincha, Ecuador. Cut flowers are among the leading exports from Ecuador, and Pedro Moncayo is known for its rose and flower cultivation. In this region, floriculture has been reported to use a variety of pesticides, including insecticides and herbicides [5,6].

In 2008, participants were predominantly recruited using the 2004 Survey of Access and Demand of Health Services (SADHS) in Pedro Moncayo County (*n* = 228). This survey was developed and administered by Fundación Cimas del Ecuador in partnership with local communities and captured a representative sample of Pedro Moncayo residents. Additional children were recruited through community leaders, local councils, and word-of-mouth (*n* = 85). The ESPINA study aimed to include a balanced mix of children who did and did not cohabitate with a floricultural or agricultural worker. The children included in the study met one of the following criteria: A) lived with a floricultural or agricultural worker for at least one year or B) never lived with a floricultural or agricultural worker, never resided in a house where agricultural pesticides were stored, and never had prior contact with pesticides.

Like in the 2008 recruitment methods, in addition to returning participants, additional participants were identified and recruited during follow-up in 2016 and 2022 via the System of Local and Community Information (SILC), previously known as the SADHS. The SILC was also developed by Fundación Cimas del Ecuador.

In 2016, a total of 554 participants aged 12–17 years were assessed, including 316 new volunteers, during two examination periods: April (FUY-8a, *n* = 331) and July to October (FUY-8b, *n* = 535). In 2022, a total of 505 participants aged 17–23 years were examined during two assessment periods: July–September (FUY-14a) and November (FUY-14b). Of the 505 participants examined in 2022 (FUY-14), 212 were returning participants who had been examined in 2016, while the remaining 293 were newly recruited at that timepoint. Across both 2016 and 2022, a total of 665 distinct individuals contributed to the dataset, with 212 individuals contributing data at both timepoints and the remaining participants providing data at only one of the two examination periods. The current analyses involved all observations in 2016 and 2022 that had measures of urinary pesticide biomarkers, which included 529 observations in FUY-8b and 505 in FUY-14a. Further details on the data collection and participant recruitment strategies have been documented in earlier publications [7,8].

### 4.1 Data collection

During the FUY-8b and FUY-14a periods, participants were examined at local schools while they were not in session. Surveys of parents (FUY-8b) and participants (FUY-14a) were conducted to capture sociodemographic and occupational information.

### 4.2 Collection of biospecimens

The participants collected urine samples upon awakening. Samples were then brought to the examination site where they were promptly aliquoted, and frozen on dry ice. The frozen urine samples were transported to Quito, Ecuador, at the end of the workday where they were stored at −70 °C (FUY8b) or −20 °C (FUY14a) for approximately 2–4 months. In 2022 (FUY-14), specific gravity was measured in fresh urine samples at room temperature in the field using a handheld refractometer prior to transport and storage on dry ice. This step ensured immediate measurement to reduce error due to temperature or freeze-thaw effects. Using a specialized courier, the frozen samples were shipped at −20 °C from Quito to the University of California San Diego (UCSD), CA, USA, where they were stored long-term at −80 °C. Samples were then shipped frozen using a specialized courier to the National Center for Environmental Health, Division of Laboratory Sciences of the Center for Disease Control and Prevention (CDC, Atlanta, GA, USA) and the Emory University’s Laboratory for Exposure Assessment and Development in Environmental Research (LEADER, Atlanta, GA, USA) for urinary pesticide biomarker quantification.

### 4.3 Urinary metabolite analysis

For FUY-8b, 19 total metabolites were measured. These included four organophosphates (3,5,6-trichloro-2-pyridinol [TCPy], malathion dicarboxylic acid [MDA], 2-isopropyl-4-methyl-6-hydroxypyrimidine [IMPy], para-nitrophenol [PNP]), three pyrethroids ([3-phenoxybenzoic acid [3-PBA], trans-3-[2,2-dichlorovinyl]−2,2-dimethylcyclopropane carboxylic acid [*trans*-DCCA], and 4-fluoro-3-phenoxybenzoic acid [4-F-3-PBA],), six neonicotinoids (acetamiprid-N-desmethyl [AND], clothianidin [CLOT], imidacloprid [IMID], 5‑hydroxy imidacloprid [OHIM], acetamiprid [ACET], and thiacloprid [THIA]), two herbicides (2,4-dichlorophenoxyacetic acid [2,4-D] and glyphosate), two fungicides (ethylene thiourea [ETU] and propylene thiourea [PTU]), and two N,N‑diethyl-meta-toluamide (DEET) insect repellents (3-[diethylcarbamoyl] benzoic acid [DCBA] and 3-[ethylcarbamoyl] benzoic acid [ECBA]). In FUY-14a the same metabolites were measured with the exception of THIA, ACET, IMID, glyphosate, ETU and PTU, and with the addition of one butenolide (flupyradifurone [FLUP]) and two sulfoximines (sulfoxaflor isomer [SLF1] and sulfoxaflor isomer 2 [SLF2]). The limits of detection (LODs) for the metabolites for each time period can be found in the Tables 1 through 4 for FUY-8b, and Tables 4 through 5 for FUY-14a. The list of urinary pesticide biomarkers measured in 2022 differed slightly from 2016 due to low detection frequencies in some analytes (e.g., IMID, ACET, THIA), budgetary constraints limiting analysis of certain compounds (e.g., glyphosate, ETU, PTU), and the inclusion of newer biomarkers (e.g., flupyradifurone, sulfoxaflor isomers) in response to evolving pesticide usage patterns in agriculture.

Quality control protocols were rigorously followed, and samples were re-extracted and re-analyzed if they failed statistical evaluation [9]. A detailed procedure for metabolite measurement has been published elsewhere [[10], [11], [12]].

### 4.4 Urinary organophosphate, pyrethroid, sulfoximine, and 2,4-D biomarker quantification

Organophosphates, pyrethroid metabolites, sulfoximine, and 2,4-D concentrations were measured through the use of liquid chromatography coupled with tandem mass spectrometry, employing isotope dilution techniques [9,13]. The quantification process involved enzymatic hydrolysis of 0.5 to 1.0 mL of urine, depending on the specific metabolite, followed by online solid-phase extraction. This procedure allows the deconjugation, extraction, and concentration of target biomarkers, which are then analyzed via reversed-phase high-performance liquid chromatography‒tandem mass spectrometry (HPLC‒MS/MS) with electrospray ionization (ESI) at the National Center for Environmental Health, Division of Laboratory Sciences of the CDC (Atlanta, GA) [9,13].

### 4.5 Urinary glyphosate quantification

Aliquots of urine (250 µL) were fortified with isotopically labeled glyphosate, diluted to a total volume of 1 mL with doubly deionized water, and subsequently extracted via a C18 solid phase extraction (SPE) method. The glyphosate was then derivatized to its heptafluorobutyl analog and concentrated for analysis. To ensure accurate measurement of urinary glyphosate, all urine samples were randomized via a Fisher-Yates shuffling algorithm before analysis to mitigate potential batch effects [14,15]. The concentrated extracts were analyzed through gas chromatography‒mass spectrometry with electron impact ionization in the multiple ion monitoring mode at the LEADER laboratory at Emory University (Atlanta, GA). The LOD was determined to be 0.25 µg/L, with a relative standard deviation (RSD) of 3 %.

### 4.6 Urinary neonicotinoid, flupyradifurone (FLUP) and DEET biomarker quantification

The method for quantifying neonicotinoid, *FLUP,* and DEET metabolites involves the enzymatic hydrolysis of 0.2 mL of urine, followed by online solid-phase extraction to release, extract, and concentrate the target biomarkers. This process is then followed by reversed-phase HPLC‒MS/MS with ESI at the National Center for Environmental Health, Division of Laboratory Sciences of the CDC (Atlanta, GA). Further details are available in Baker et al. (2019) [9]. The precision of these measurements, indicated by the RSD of multiple analyses of two urine-based quality control (QC) materials, was under 6 %, depending on the specific biomarker and its concentration.

### 4.7 ETU and PTU quantification in urine

To minimize any potential batch effects, all samples were randomized via a Fisher–Yates shuffling algorithm before analysis [14,15]. An 800 µL urine aliquot was spiked with isotopically labeled internal standards and 50 µL of 2.2 N hydrochloric acid. The samples were then processed using an absolute solid‒liquid extraction (SLE) cartridge. The resulting eluates were dried and reconstituted with 100 µL of 0.1 % formic acid. These extracts were analyzed via HPLC‒MS with ESI at the LEADER laboratory at Emory University (Atlanta, GA). ETU, PTU, and their labeled analogs were quantified in the multiple ion monitoring mode [16]. The concentrations of ETU and PTU were determined via isotope dilution calibration. The LODs for ETU and PTU were 0.625 and 1.63 µg/L, respectively, with an RSD of 7 %.

### 4.8 Imputation for values below the LOD

Except for TCPY and PNP, which have 100 % detection rates (>LODs), values below the LOD were imputed as the LOD/√2. Glyphosate, ETU, and PTU had trace concentrations detected, even thought they were below the LOD. For these three metabolites, we used these machine derived values if non-zero instead of imputation. Machine-read values were used for pesticide analysis, as they were available for all measurements, including those recorded as zero. Machine read values were available for observations below the LOD for 328 observations (62.00 %) for PTU, 48 observations (9.07 %) for ETU, and 69 observations (13.04 %) for glyphosate. A total of 147 observations (27.79 %) for PTU and 51 of observations (9.64 %) for ETU were below the LOD, but did not have machine derived values. This approach reflects an assignment-based imputation strategy (LOD/√2) and aligns with established practices for handling non-detects in biomonitoring data.

### 4.9 Urinary creatinine quantification

For the FUY-8b cross-section, urinary creatinine concentration was measured to account for urinary dilution via HPLC‒MS/MS with ESI. A 10 µL aliquot of urine was diluted before analysis [17], with no further sample preparation. The LOD was 5 mg/dL, with an RSD of 7 %. Measurements were conducted at the LEADER laboratory at Emory University (Atlanta, GA).

### 4.10 Urinary specific gravity quantification

In FUY14a, specific gravity was measured with an ATAGO PAL-10S digital refractometer from ATAGO (ATAGO Co., Ltd., Tokyo, Japan.) in fresh urine samples at room temperature, following standard procedures. To enable comparisons between FUY-8b and FUY-14a, we estimated urinary specific gravity for FUY-8b by converting urinary creatinine values using a formula that accounted for age and sex, derived from a nationally representative sample of US children and adolescents [18].

### 4.11 Statistical analysis

Participant characteristics were calculated using means for normally distributed variables and percentages for categorical variables (Table 1). Geometric means were calculated if the frequency of detection for that analyte was above 30 %. In addition to the geometric mean and 95 % confidence interval (95 % CI), we calculated percentile values (i.e., 10th, 25th, 50th, 75th, 90th and 95th) for urinary biomarker concentrations for FUY-8b (Table 1) and FUY-14a (Table 2). The detection rate ( % detectable) was calculated by dividing the number of samples that had concentrations above the LOD by the total number of samples minus observations that had interfering substances. Despite having machine-derived trace concentrations for select observations below the LOD for ETU, PTU and glyphosate, the percentage detectable included only all observations above the LOD in the numerator. In addition to wet weight (Tables 1 & 4), metabolite concentrations were adjusted for creatinine for FUY-8b (Table 2), and specific gravity adjustment was performed for both FUY-8b and FUY-14a. (Table 3, Table 5, respectively). Metabolites for FUY-8b were standardized for creatinine by dividing wet weight values by urine creatinine concentration to obtain metabolites in micrograms per gram (µg/g) of creatinine. Urinary pesticide biomarker concentrations were adjusted for calculated specific gravity for FUY-8b and measured urine specific gravity for FUY-14a by multiplying the wet weight concentrations by $\frac{Specific\;gravity_{mean}-1}{Specific\;gravity-1}$.

The data show detection rates of 30 % and above for 11 out of 19 metabolites measured in 2016 and 12 of 16 metabolites measured in 2022. In both periods, two organophosphate pesticide metabolites, 3,5,6-trichloro-2-pyridinol (TCPY) and para-nitrophenol (PNP), had a 100 % detection rate. These data provide evidence of pesticide exposure in participants in agricultural communities, highlighting the need for continued monitoring and interventions to mitigate exposure risk.

## Limitations

One major limitation of measuring pesticide metabolites in urine using mass spectrometry is the short half-life of metabolites, which leads to a narrow window of exposure. Since pesticides are often rapidly metabolized and excreted [19], a single urine sample may not accurately reflect long-term exposure, requiring repeated sampling. Additionally, inter-individual differences in metabolism and excretion can significantly affect measured concentrations. Factors such as age, genetics, hydration status, and kidney function influence how quickly pesticide metabolites appear and are cleared from urine [20]. While most pesticide biomarkers presented are specific to pesticide exposures, the metabolite 3-PBA is a common metabolite of multiple pyrethroid pesticides, making it difficult to determine specific exposure sources. Furthermore, there is no universal reference standard for many pesticide metabolites, complicating result interpretation; nonetheless, biomonitoring data of a representative population can allow for exposure level comparisons across different populations.

## Ethics Statement

The ESPINA study received approval from the Institutional Review Boards at the University of Minnesota, the University of California San Diego (Approval #160,060), Universidad San Francisco de Quito (Approval #2016–047E), UTE University (Approval # CEISH-2021–001), and registered in Ecuador's Ministry of Public Health. Additionally, our study was endorsed by the local governments of Pedro Moncayo County. Informed consent of adult participants was obtained, in addition to parental permission for participation and child assent for participants who were minors.

## CRediT authorship contribution statement

**Rajendra P. Parajuli:** Conceptualization, Data curation, Investigation, Methodology, Writing – original draft. **Briana N.C. Chronister:** Formal analysis, Investigation, Methodology, Writing – review & editing. **Dana Boyd Barr:** Writing – review & editing. **José Ricardo Suárez-López:** Conceptualization, Funding acquisition, Project administration, Resources, Software, Methodology, Supervision, Writing – review & editing.

## Data Availability

Mendeley DataUrinary pesticide biomarkers from adolescence to young adulthood in an agricultural setting in Ecuador: ESPINA study 2016 and 2022 examination data. (Original data).
